# Training associated alterations in equine respiratory immunity using a multiomics comparative approach

**DOI:** 10.1038/s41598-021-04137-3

**Published:** 2022-01-10

**Authors:** Anna E. Karagianni, Dominic Kurian, Eugenio Cillán-Garcia, Samantha L. Eaton, Thomas M. Wishart, R. Scott Pirie

**Affiliations:** The Roslin Institute and Royal (Dick) School of Veterinary Studies, University of Edinburgh, Easter Bush, Midlothian, EH25 9PS UK

**Keywords:** Biological techniques, Biotechnology, Cell biology, Computational biology and bioinformatics, Immunology, Molecular biology, Diseases

## Abstract

Neutrophilic airway inflammation is highly prevalent in racehorses in training, with the term mild to moderate equine asthma (MMEA) being applied to the majority of such cases. Our proposed study is largely derived from the strong association between MMEA in racehorses and their entry into a race training program. The objectives of this study are to characterise the effect of training on the local pulmonary immune system by defining the gene and protein expression of tracheal wash (TW) derived samples from Thoroughbred racehorses prior to and following commencement of race training. Multiomics analysis detected 2138 differentially expressed genes and 260 proteins during the training period. Gene and protein sets were enriched for biological processes related to acute phase response, oxidative stress, haemopoietic processes, as well as to immune response and inflammation. This study demonstrated TW samples to represent a rich source of airway cells, protein and RNA to study airway immunity in the horse and highlighted the benefits of a multiomics methodological approach to studying the dynamics of equine airway immunity. Findings likely reflect the known associations between race-training and both airway inflammation and bleeding, offering further insight into the potential mechanisms which underpin training associated airway inflammation.

## Introduction

During the early phase of training, racehorses commonly develop airway inflammation. Prevalence rates as high as 70–80% have been reported^1–4^, and generally reduce as training progresses^5,6^. This airway inflammatory response, when associated with poor athletic performance and/or chronic coughing has been termed Mild to Moderate Equine Asthma (MMEA)^4,7^. Although the precise aetiopathogenesis of MMEA remains undefined, proposed aetiological candidates have included both infectious (bacteria and viruses) and non-infectious (organic dust) agents; indeed, some cases may be attributable to a combination of infectious and non-infectious factors acting in concert^4^. In relation to infectious factors, the reported association with the early training period may reflect an initial period of susceptibility with the subsequent temporal establishment of immunity. Although entry into race training may result in increased exposures to airborne infectious and non-infectious agents, it is also associated with a significant increase in exercise intensity and frequency. The well recognized association between high intensity exercise and symptoms of respiratory infection amongst human athletes has fueled interest in the impact of training on immune function^8,9^. Despite localization of inflammation to the airways in MMEA^7^, few studies have assessed the influence of training on immune cell function specifically at this anatomical site, a key consideration in light of the recently reported disassociation between the response of blood derived equine monocytes and alveolar macrophages (AMs) to training^10–13^. Both an exercise-associated reduction in equine AM phagocytic capacity^14^ and a training-associated derangement in the responsiveness of equine AMs to various TLR ligands have been reported, theoretically reflecting increased susceptibility to opportunistic infection^10^. In agreement, we recently demonstrated a training-associated alteration in equine AM basal gene expression, and confirmed a degree of immunomodulation at the level of the airways^12^. Consequently, there appears to be sufficient justification for further defining the link between training and airway immunity.

This study was designed to further expand upon and adapt our previously published observations, through targeting TW-derived cells from a larger number of horses and adopting a more rigorous study design and extensive analytical approach (RNAseq and proteomics). The adopted analytical approach was selected primarily to further define the mechanisms underpinning training-associated immunomodulation in racehorses, with the added potential of allowing further characterization of training-associated airway diseases and identify disease- and/or disease susceptibility-associated biomarkers and/or novel therapeutic targets.

Thus, the principle objective of the study was to investigate the impact of entry into race training on airway immune function. The “open window” theory, reflecting a temporal association between intense training and increased susceptibility to opportunistic infection, is well recognized within the field of human exercise immunology^8,15^ and may explain the association between increased tracheal mucus (a marker of MMEA) and both time in training and the isolation of *Streptococcus zooepidemicus* and non-haemolytic streptococci from respiratory tract secretions of racehorses in training^6^. Such supportive information has the capacity to inform management practices which, in light of the inherent necessity for episodes of high speed exercise within a training regimen, are likely to focus on either opportunistic pathogen avoidance schemes and/or novel immunomodulation strategies.

We hypothesised that intense training has a significant impact on the respiratory immune response of racehorses and that a combined transcriptomics and proteomics approach would further elucidate the specific immunological downstream molecular cascades impacted by this activity. In order to address the above hypotheses, we studied the gene expression profile of tracheal wash (TW)-derived cells and the proteomic profile of whole TW samples derived from UK national hunt Thoroughbreds, both before and after entry into a training schedule. Furthermore, as the racing Thoroughbred offers an ideal model for the study of the impact of exercise it was considered that the results of this study may have significant translational application to the increasing body of evidence relating to human exercise immunology.

## Results

### Cell recovery and populations

**Figure 1:**
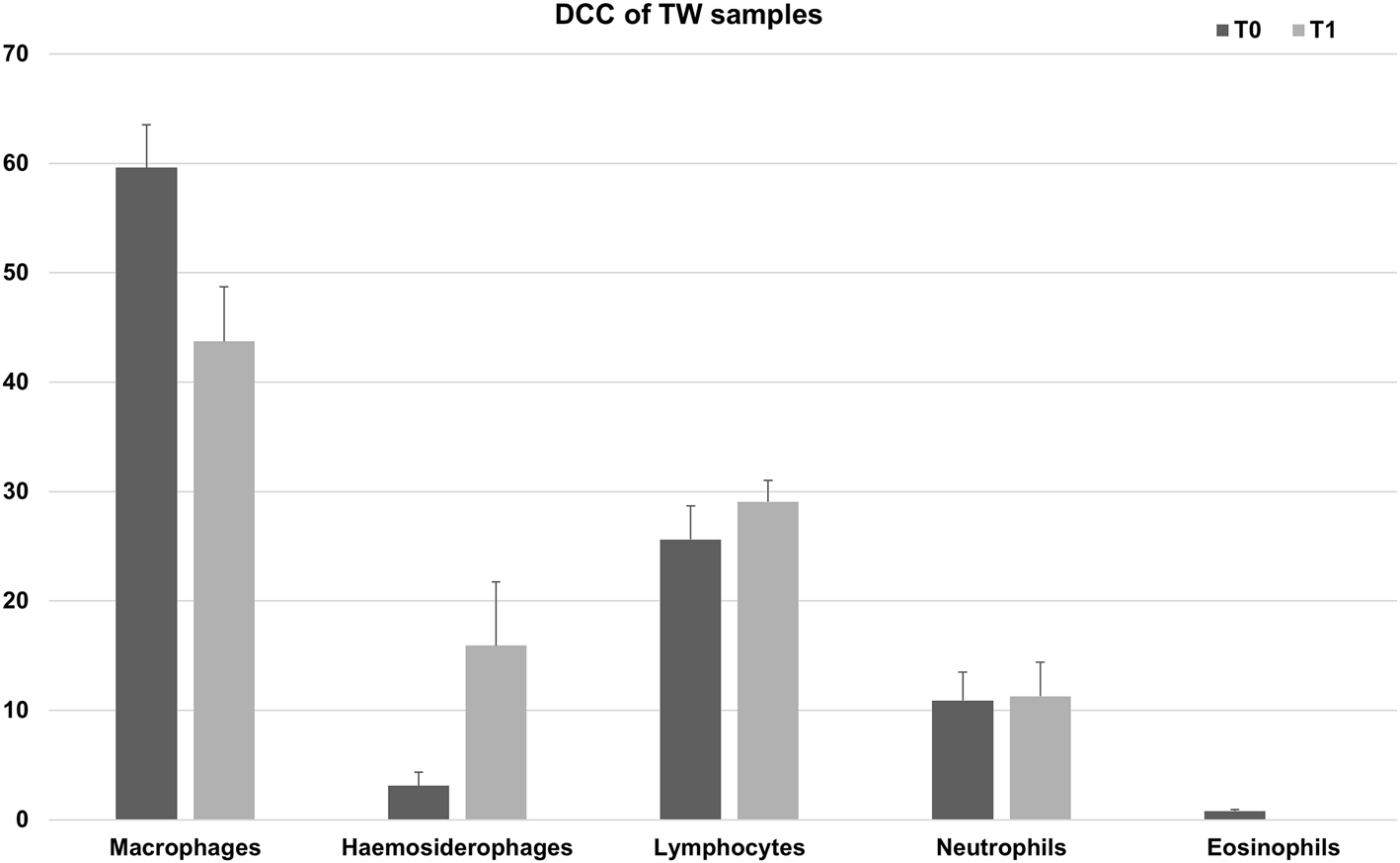
Tracheal wash cytology. Percentages (mean + SEM %) of macrophages, haemosiderophages, lymphocytes, neutrophils and eosinophils in the TW samples of 16 Thoroughbred racehorses during resting (T0) and training (T1) period. Note, the macrophage ratio represented the ratio of macrophages without phagocytosed haemosiderin; consequently, the decrease in the ratio of macrophages at T1 reflected the increase in the proportion of macrophages containing haemosiderin (i.e. haemosiderophages) at this time point.

The present study used a series of 32 horse-derived TW samples from 16 Thoroughbred racehorses at two time points (T0-resting period, T1-training period) and an average of 10.8 × 10^6^ (± 1.9) cells per sample were isolated, consistent with previously reported data^16,17^. None of the animals had clinical signs of respiratory disease or a history of poor performance. Results of the differential cell counts (DCC) are shown in Fig. 1. Although a slightly greater neutrophil and lymphocyte percentage was detected in samples obtained from horses during the training period, there was no significant difference between groups for all immune cells in the DCC. As expected, a remarkable increase of haemosiderophages was reported during training. Since all samples were collected from racehorses, the presence of haemosiderophages in these samples was predictable, as almost all racing Thoroughbred horses will bleed to varying degrees into the airways^18^.

### Intense training-induced gene expression in equine tracheal wash derived cells

**Figure 2:**
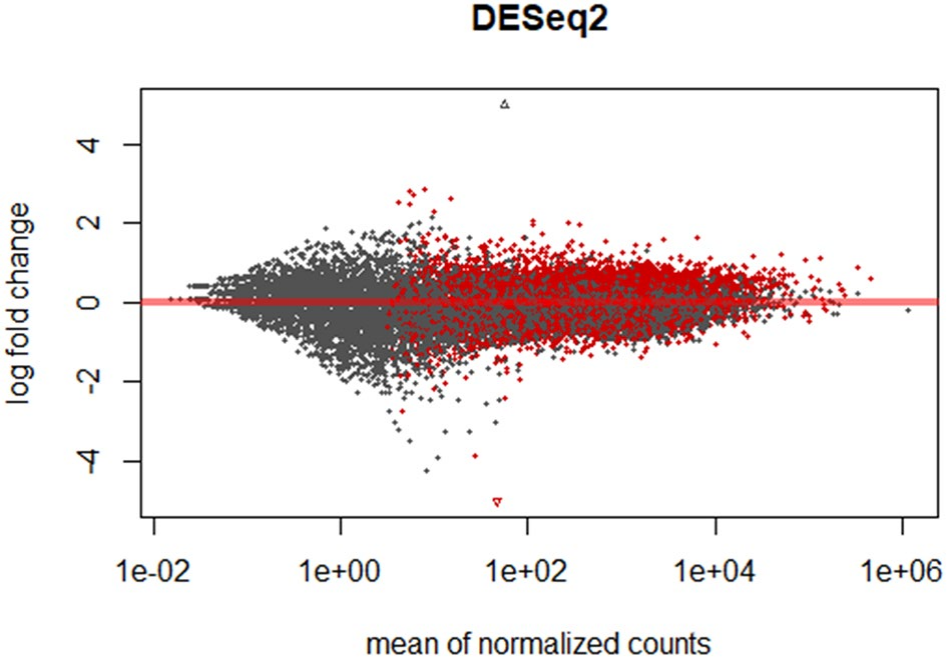
MA plot. The gene expression data visualised as a two-dimensional scatter plot of the log2 ratio of expression values between the two timepoints**.** MA plots show the log-fold change (y axis) (M-values, i.e. the log of the ratio of level counts for each gene between T1 and T0) against the log-average (x axis) (A-values, i.e. the average level counts for each gene across the samples). Each dot represents one gene, and the red colour indicates the 2138 genes identified to be differentially expressed between the two time points using a false discovery rate (FDR) of < 0.05.

**Figure 3:**
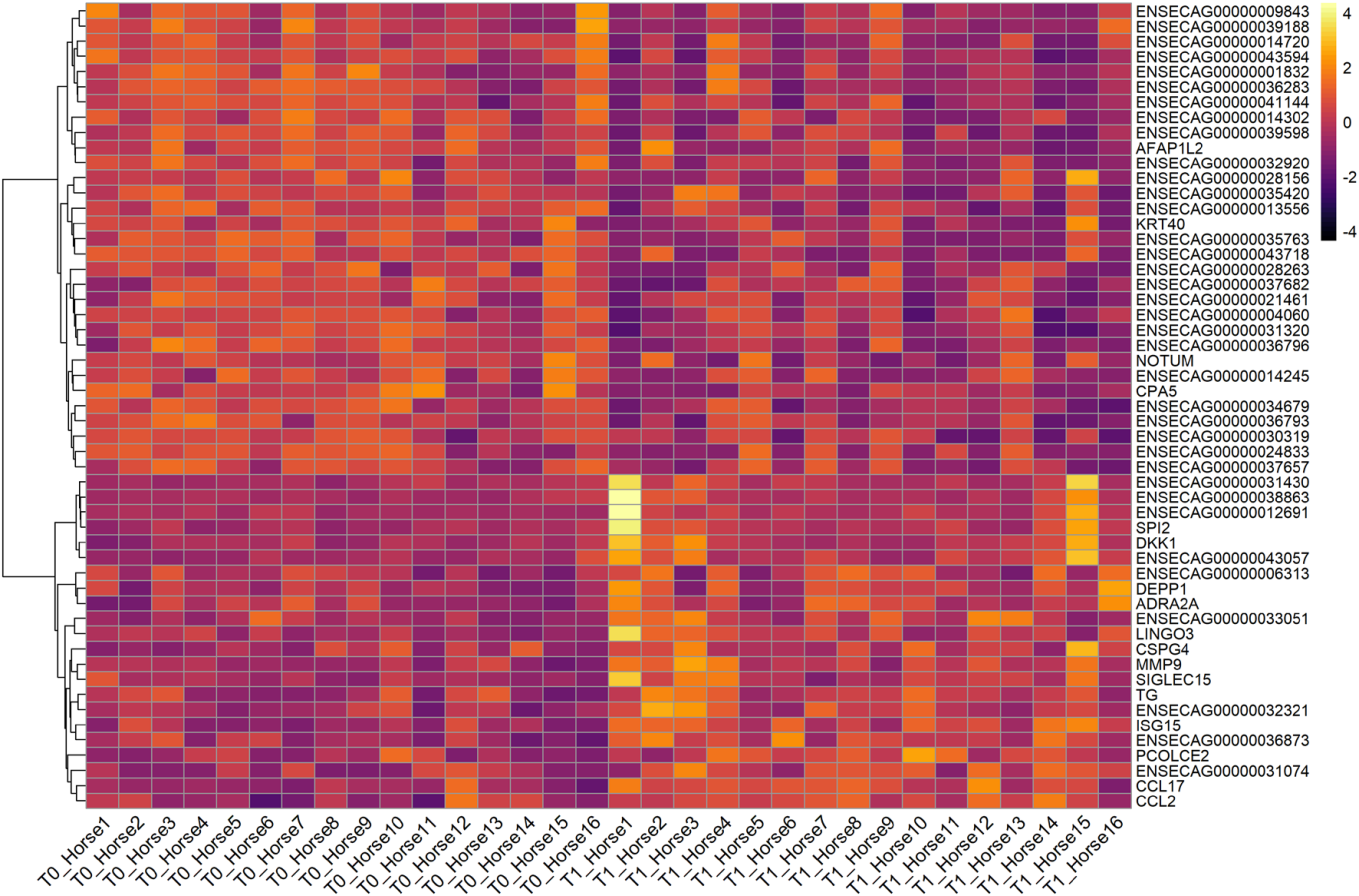
Gene expression of tracheal wash derived cells is modified during intense training. Hierarchical clustering based on normalized gene counts of differentially expressed genes in samples derived from 16 Thoroughbred racehorses at two time points using a false discovery rate (FDR) of < 0.05 and a fold change of 1.5.

Whole-transcriptome profiling (RNAseq) was performed on TW derived cells from 16 Thoroughbred racehorses prior to (T0) and approximately 2.5 months following entry into an intense training program (T1). A total of 21,357 equine genes were identified, and the reads were quantified to identify those that were differentially expressed between the two timepoints (Fig. 2). Intense training produced a radical change in equine gene expression, with 2138 differentially expressed genes (1122 upregulated and 1016 downregulated) being identified between the two time points. We defined differentially expressed genes to be those showing up- or downregulation following intense training with a false discovery rate (FDR) below 0.05. The complete lists of mapped genes and differentially expressed genes are presented in the supplemental material (Supplementary Data 1). Figure 3 summarizes the differentially expressed genes using a false discovery rate (FDR) of < 0.05 and a fold change of 1.5. These included chemokines related to immunoregulatory and inflammatory processes (*CCL2*, *CCL17*), genes related to cell proliferation (*AFAP1L2*), signal transduction (*NOTUM*, *DKK1*, *CSPG4*), immunomodulation (*SPI2*, *SIGLEC15*), oxidative stress (*DEPP1*), tissue remodelling (*MMP9*, *PCOLCE2*), as well as IFN stimulated genes (*ISG15*). Selected genes which were evaluated by qPCR confirmed their differential expression (Supplementary Data 2).

### Intense training-induced protein expression in equine tracheal wash samples

In order to expand on the RNA studies, we also defined the airway protein profiles (total proteome) of TW samples, thus revealing the mechanisms which may underpin any alterations in immune function. Protein extraction was successfully performed on TW samples from 13 out of 16 horses. Insufficient protein was isolated from TW samples derived from Horses 7 and 12 at T0 and Horse 3 at T1; thus, these animals were excluded from the proteomic analysis at both time points. An average of 1.6 ± 0.2 (+ SEM) mg protein was isolated from 500 µL of TW per animal. Twenty micrograms per sample were pooled for each time point and samples were processed as described above. In order to visualise the total protein load of all the samples, samples were run on gradient gels and stained with instant blue protein stain, as previously described^17^. Subsequently, protein samples were submitted for proteomic analysis. We identified distinctive protein signatures in the Thoroughbred TW samples in response to training. In total, 802 unique proteins were detected, 260 of which were identified as differentially expressed, based on arbitrary cut-off values of ≥ 1.2-fold change for the upregulated proteins and ≤ 0.8-fold change for the downregulated proteins. Applying these criteria, 103 proteins were upregulated and 157 were downregulated. The complete lists of proteins are presented in the Supplementary Data 3. The 30 proteins with the greatest differential expression in the TW samples during the training period (time point T1), are shown in Supplementary Data 3. These protein’s functions were related to immune response and lung biology and included pulmonary surfactant-associated proteins (SFTPA1, SFTPB), complement factors (CA6, C1R), heat shock proteins (HSPE1), antigen presentation molecules (DRA, PIP), antioxidants (PRDX3, SOD2), haemopoietic factors (HBE1, HBA, FGA, TFRC), protein synthesis (PSMA5, HSPE1, PHB) and apoptosis related factors (GSN). Thirty percent of the proteins detected here were also previously detected from TW samples derived from racehorses^17^.

### Functional analysis of RNAseq and proteomic datasets

In line with previous observations^19^, the correlation between individual molecular immune transcripts and proteins was low. Interestingly, although analysis of both the RNAseq and proteomic datasets revealed a limited number of gene/proteins with a similar differential expression in response to training (Supplementary Data 3), a clear correlation between the two complete datasets was apparent at both the pathway and cascade level. KEGG pathway and gene ontology enrichment analysis of the upregulated and downregulated genes (Supplementary Data 4) and proteins (Supplementary Data 5) was performed using the Database for Annotation, Visualization, and Integrated Discovery (DAVID) annotation software (https://david.ncifcrf.gov/^20^). These analyses revealed a training associated activation (at the level of both gene and protein expression) of pathways and biological processes involved in acute inflammatory response, immune defence and immune cell activation and differentiation. Additional pathways which were activated in response to training included metabolic pathways, cellular and oxidative stress-associated pathways and pathways related to heme degradation or haematopoietic abnormalities; these latter pathways likely reflected the higher prevalence of EIPH in horses in training. Importantly, in the RNAseq dataset, we detected pathways related to viral infection and interferon signalling.

**Figure 4:**
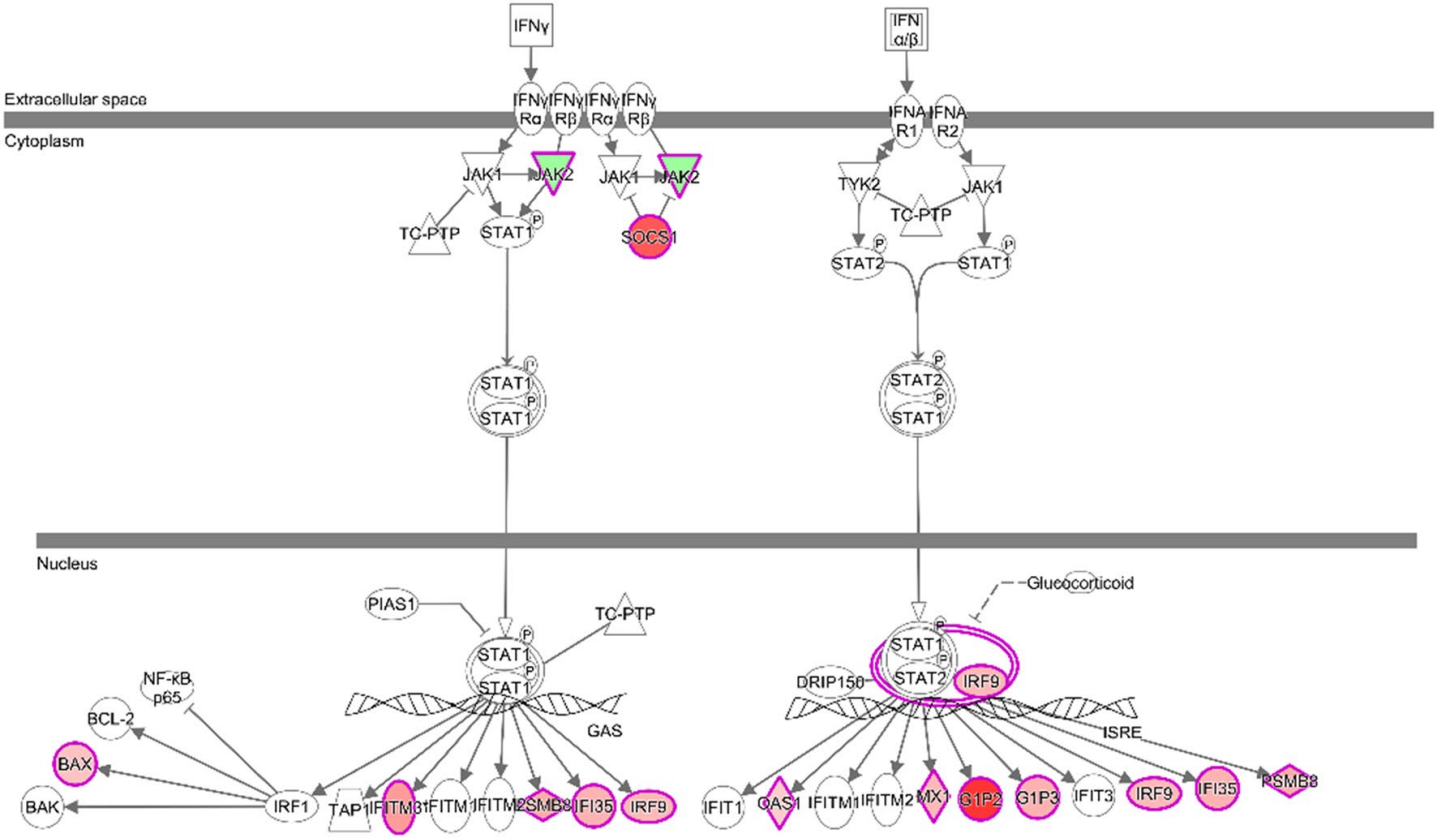
Ingenuity^®^ Pathway Analysis predicted signs of activation of IFN signalling pathway. Ingenuity^®^ Pathway Analysis (IPA) predicted signs of activation of IFN signalling pathway in relation to intense training (z-score = 1.897 and p value = 7.28E−05). A canonical pathway network was derived using IPA by screening 2138 differentially expressed genes in tracheal wash (TW) samples derived from Thoroughbred racehorses before and during training for their impact on IFN signalling. Green shades indicate relative downregulation of genes during training, while red shades indicate relative upregulation. More intense (darker) colours indicate greater increases or decreases.

**Figure 5:**
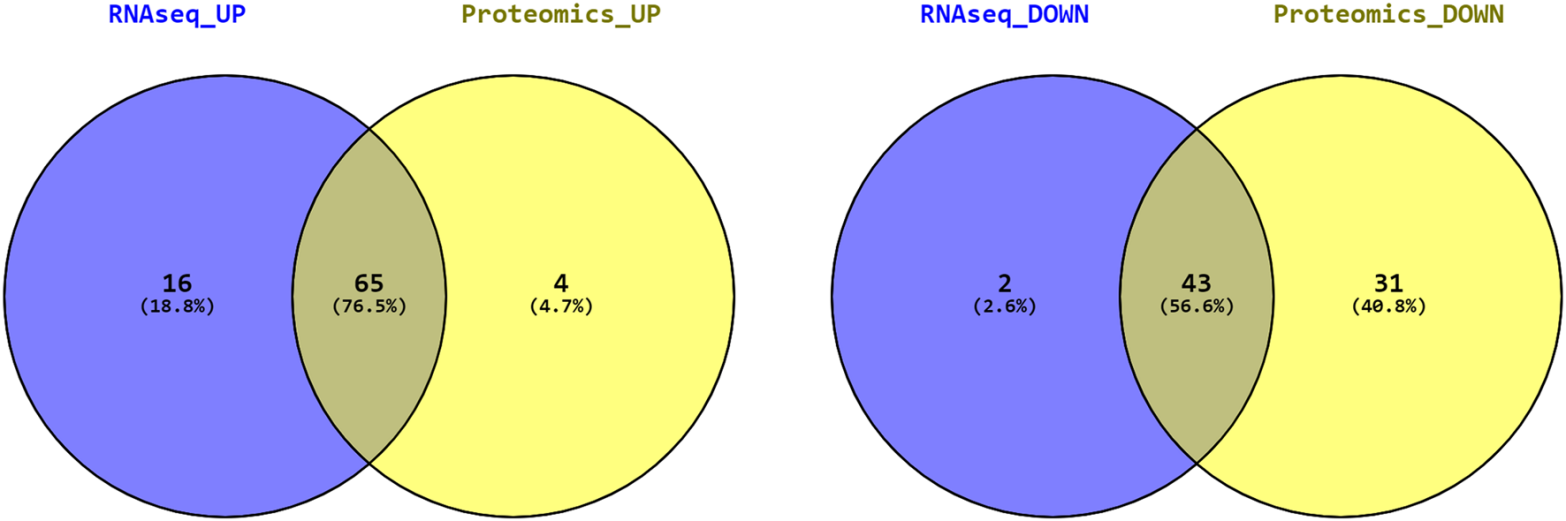
Comparative analysis of main diseases and biofunctions associated with differentially expressed genes and proteins. Comparative analysis of main diseases and biofunctions associated with upregulated and downregulated genes and proteins in response to intense training. Venn diagrams were generated based on the IPA Disease and Biofunction results derived from the upregulated (left panel) and downregulated (right panel) transcriptomic and proteomic datasets.

Ingenuity Pathway Analysis (IPA) was also used to functionally assess the list of differentially expressed genes and proteins^21^. Canonical pathway network analysis based on the 2138 differentially expressed genes indicated activation of IFN signalling (Fig. 4). In line with DAVID analysis, cellular stress-related functions, such as oxidative phosphorylation and mitochondrial dysfunction, as well as those related to the immune response and inflammation were identified as being highly enriched. Interestingly, despite the small overlap between the transcriptomic and proteomic datasets the majority of the Diseases and Biofunctions determined to be enriched by IPA were common between the two datasets; 76.5% of the upregulated and 56.6% of the downregulated molecules (Fig. 5). Full database content for each IPA category and the genes related to this are presented in Supplementary Datas 6 and 7.

#### Training-associated immune response on equine respiratory immunity

In line with previous literature in different species, including humans^8^ and horses^10,12^, the current study also supports the principle that the immune system is very responsive to exercise. Functional annotation of the transcriptomic and proteomic datasets revealed a strong association with different immune system processes, including response to infection. These included functions such as antigen presentation (*HLA-DMA*, *HLA-DMB*, *PSMB8*, *PSMB9*, *PSMB10*, *PSME1*, *PSME2*, *TAPBPL*, *DRA*), complement activation, immune cell chemotaxis (neutrophil, monocyte and lymphocyte), viral responses and regulation of IFN signalling (Supplementary Datas 1, 3–5).

**Figure 6:**
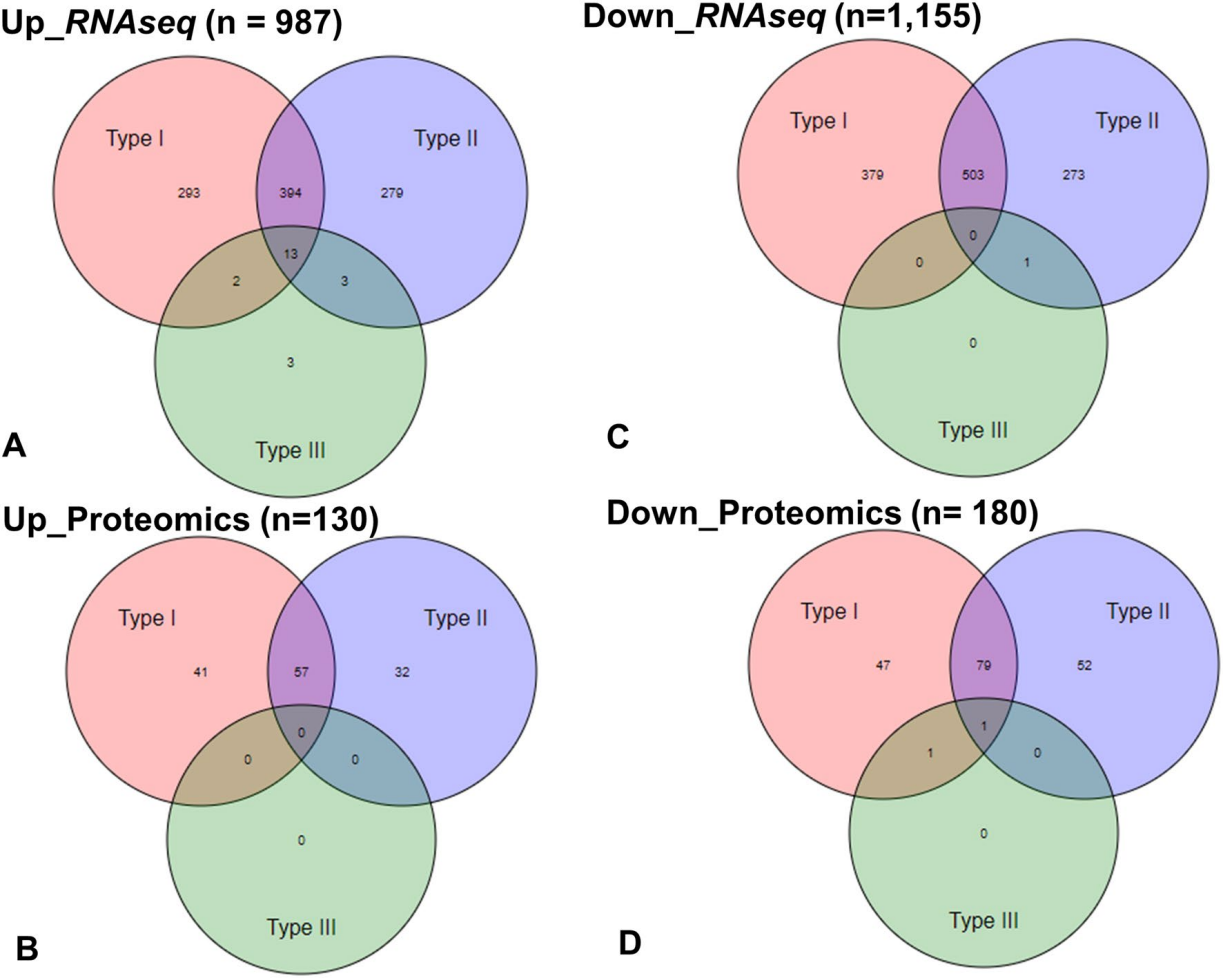
Venn diagram of genes and proteins regulated by IFN types. The Venn diagram shows the number of genes or proteins regulated by one or more IFN type (Type I, II or III). Venn diagrams were generated based on the upregulated genes (**A**), proteins (**B**) and the downregulated genes (**C**), proteins (**D**) during the training period (interferome database). Note there are very few datasets for type III interferons—only twenty datasets from just two human experiments; thus any interpretation of type III interferons should be done with caution (http://www.interferome.org/interferome/home.jspx).

Interferons have diverse functional properties and are involved in numerous biological processes related to host response to infection, inflammation, cancer, autoimmunity and metabolic disorders. Based on the publicly available interferome database (http://www.interferome.org/interferome/home.jspx), we detected the differential expression of several interferon regulated genes and proteins during intense training^22^. Figure 6 shows the Venn diagrams created based on the number of genes or proteins regulated by one or more IFNs (Type I, II or III).

Many of the recently described panel of 90 highly conserved mammalian type 1 interferon-stimulated genes (ISG), were differentially expressed in our data^23^; these genes and a few encoding proteins, most of which were upregulated are presented in Table 1. They are broadly involved in antigen presentation, antiviral response, IFN activation and repression, apoptosis and cell signalling.

**Table 1:** Core IFN stimulated genes and proteins differentially expressed during training.

Antigen presentation genes and proteins↑	Antigen presentation genes and proteins↓
*HLA-DMA*	ERAP1
*HLA-DMB*	PSMA5
*PSMB8*	
*PSMB9*	
*PSMB10*	
*PSME1*	
*PSME2*	
*TAPBPL*	
*DRA,* DRA	

Antiviral genes and proteins↑	Antiviral genes and proteins↓
*ISG15*	*MORC3*
*ISG20*	
*MX1*	
*OAS1*	
*RSAD2*	
*SHISA5*	

PAMP sensing—IFN pathway genes and proteins↑	PAMP sensing—IFN pathway genes and proteins↓
*IRF9*	*AZI2*
*DHX58*	

IFN suppression genes and proteins↑	IFN suppression genes and proteins↑
*IFI35*	
*SOCS1*	
*TRAFD1*	
*TRIM21*	

Cell signalling + apoptosis genes and proteins↑	Cell signalling + apoptosis genes and proteins↓
*TNFSF10*	*RICTOR*

Miscellaneous genes and proteins↑	Miscellaneous genes and proteins↓
*CMPK2*	*FMR1*
*DNAJA1*	
*SERTAD1*	
*XAF1*	
C2	

Interestingly, the classical sensor for RNA PAMPs, *DHX58* was upregulated, whereas, the DNA sensor *AIM2* was downregulated. The fragile X mental retardation (*FMR1*) gene that encodes an RNA-binding protein that plays a role in intracellular RNA transport and in the regulation of translation of target mRNAs, was downregulated in response to training. *FMR1* has been recently linked to the IFN response and has been shown to be a proviral factor for influenza virus and was found to induce mild restriction of HIV-1^24,25^. Other host proviral genes (*TOR1B*, *IFI44*, *DNAJA1*, *AREG*) and LCN2 protein involved in influenza A virus replication in mice were also upregulated during training in TW derived cells^25^.

Vital IFN-induced antiviral factors such as *MX1* and *RSAD2*, as well as *IRF9* (a key transcription factor involved in IFN induction and response) were also upregulated in relation to intense training. Moreover, a link between local synthesis of early components of the complement system and the type I IFN response is now well documented^23^. Complement components such as C1r and C2 proteins were also upregulated in TW samples during the training period. In line with this, protein SERPING1 a negative regulator of C1r was downregulated in the same samples. On the other hand, other members of ISG that play a role in the suppression of the IFN system, such as *IFI35*, *TRAFD1*, *TRIM21* and *SOCS1* were also upregulated. The negative regulation of the IFN response is multifaceted and necessary in order to avoid excessive/perpetual activation of IFN-induced pathways^23^.

Other pathways involved in immune processes, such as immunological response, respiratory disease and inflammation were downregulated (Supplementary Data 4, 5, 7). Based on the interferome dataset the majority of the interferon regulated genes seem to be downregulated in both datasets (1155 in RNAseq and 180 in proteomics) during training (Fig. 6). These results revealed key signalling networks involved in Natural killer cell mediated cytotoxicity, T and B cell receptor signalling pathway and TNF signalling. For example, several components such as *CD69*, *NFAT5*, *NFKBIZ*, *NKAP*, *TAGAP*, involved in T cell activation and differentiations, as well as the antimicrobial factor *LYZ* and the pro-inflammatory cytokine TNF were significantly downregulated. Besides, the anti-inflammatory elements *TIGIT*, a T cell suppressor, as well as *SOCS1*, a negative regulator of cytokine signalling were upregulated. Thus, there seems to be some evidence of training associated immunosuppression on equine TW derived cells.

### Transcriptomic profile of racehorses with high neutrophil count on TW samples

**Figure 7:**
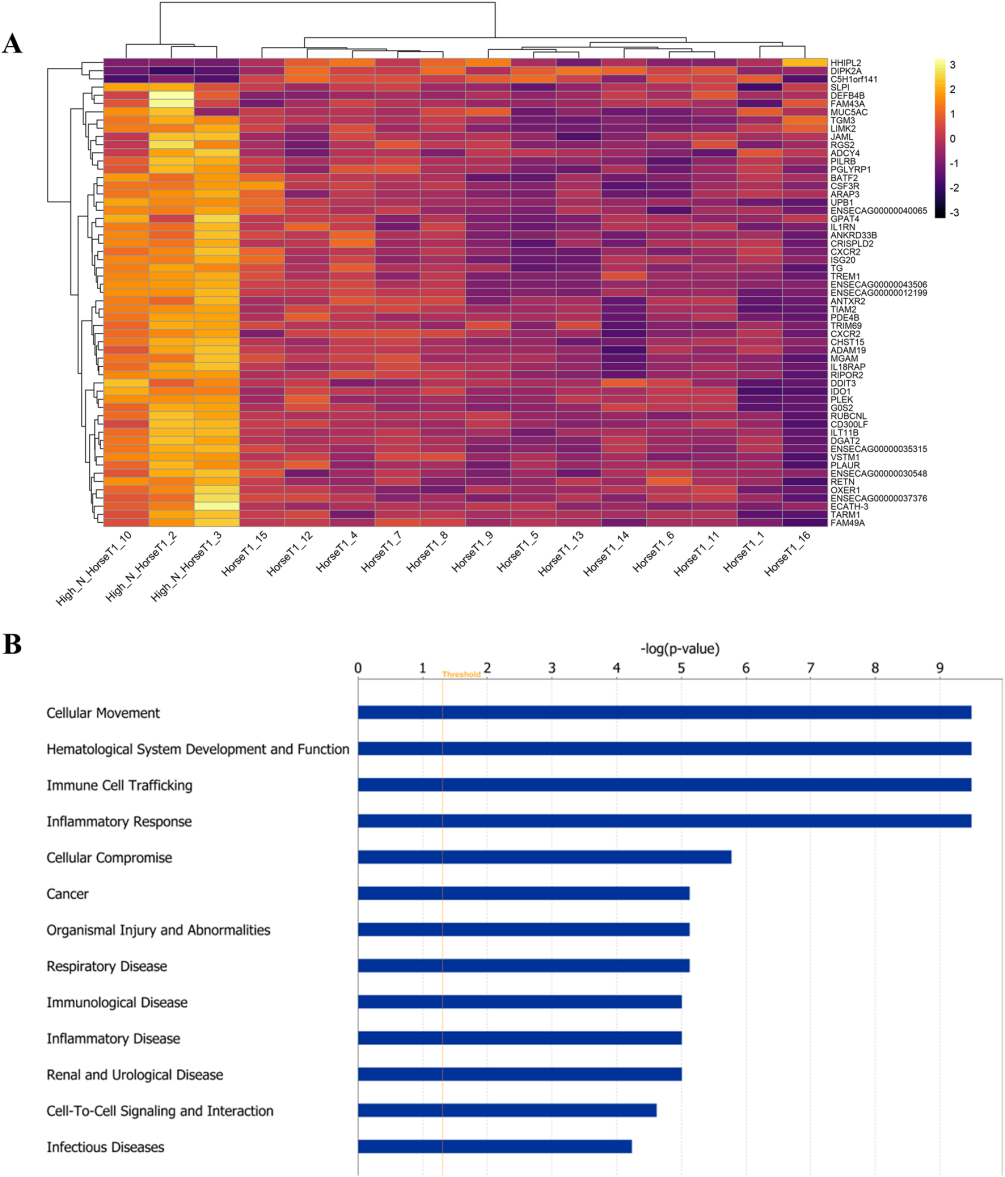
Transcriptomic profile of racehorses with high neutrophil count on TW samples. (**A**) Hierarchical clustering was based on normalized gene counts of differentially expressed genes in samples derived from horses with and without a neutrophil count (High_N) exceeding publicised acceptable limits (54 were upregulated and 3 were downregulated). (**B**) Immune response-related biofunctions were enriched in airway cells derived from Thoroughbreds with high neutrophil count.

Despite the high prevalence of MMEA in racehorses, that can be as high as 80% in some cohorts^1,2^, only Horses 2, 3 and 10 were detected with neutrophil proportions beyond publicised acceptable limits during the training period (High_N group), based on the TW DCC cut off values: neutrophils > 20%^7,26,27^. Furthermore, almost all horses apart from five (Horse 4, 9, 10, 11, 16) were affected with EIPH, based on the presence of haemosiderophages in their TW samples. Since none of the horses displayed any clinical signs, the forms of both conditions were considered to be subclinical. In an attempt to further dissect the effect of the neutrophil infiltration on the gene expression of these animals, whole genome analysis was performed on TW samples derived from Horses with high neutrophil numbers (High N, n = 3) versus healthy individuals/controls (n = 13). Based on RNA sequencing data we identified 57 equine genes (Fig. 7A) differentially expressed in the High N group compared to the controls (Supplementary Data 8) (following multiple-testing correction, false discovery rate (FDR) p-value < 0.05). These included genes related with neutrophil migration (*CSF3R*, *JAML*, *PDE4B*, *TREM1*), immune system process (*BATF2*, *CSF3R*, *IDO1*, *ISG20*, *JAML*, *PGLYRP1*, *PDE4B*, PLEK, SLPI, TREM1), cytokine production (*DDIT3*, *IDO1*, *PGLYRP1*, *PDE4B*, *TREM1*) and cell chemotaxis (*CSF3R*, *JAML*, *PDE4B*, *TREM1*) (Supplementary Data 8).

To gain a global view of the results, functional analysis was performed as previously described using IPA and DAVID in both datasets. As expected, in samples derived from animals affected with high neutrophil counts various relevant biological processes related to neutrophil infiltration were observed. Examples are neutrophil and granulocyte migration, neutrophil and granulocyte chemotaxis and neutrophil extravasation. Other immune related terms were also detected; response to biotic stimulus, defence response, response to external stimulus, immune system process, inflammatory disease/response, response to stress and regulation of response to stimulus (Fig. 7B, Supplementary Data 8).

## Discussion

To our knowledge, this is the first study to report on the “effect of training” on UK Thoroughbred national hunt racehorses, determined by both transcriptomic and proteomic analysis of TW samples harvested before and during a period of training, thus permitting an assessment on the basal gene expression of principal innate immune cells of the equine airway. TW samples are routinely collected in contrast to the less readily available and more technically invasive bronchoalveolar lavage (BAL)-derived samples, which have previously been favoured for transcriptomic and proteomic analyses^28–30^. The transcriptome was derived from high throughput sequencing, and results were analysed with a paired design to account for individual variation. Animals were of similar age, same sex and breed and lived in a similar environment. All animals recruited in this study were from the same yard and subject to the same general management structure. RNA-seq data were analysed using DEseq2 software, a widely used tool in high-throughput RNA sequencing studies using raw counts^31^. Compared with a single-end strategy, the paired-end strategy used in this study has been recommended as it minimises the likelihood of false positives^32^; moreover, the validity of the data, in terms of differential expression, was supported by the qPCR results. However, irrespective of the methodology used, considering the genetic variability among individuals and the limited sample size, the potential for false-positive results remains, necessitating caution when interpreting individual gene expression data in RNAseq-based studies. Similar caution should be exercised when interpreting the differential expression of individual proteins in the proteomic dataset. Thus, the consideration of linked, rather than individual, genes and proteins conveys more confidence when interpreting these kind of data^32^.

Our experimental protocol allowed us to investigate the effect of training on airway innate immune responses at both the gene and protein level. Although this revealed significantly limited overlap between the RNAseq and proteomic datasets, this was not unexpected as such a lack of concordance between gene and protein expression has been well documented^33,34^ and can be attributed to a variety of factors such as post-transcriptional machinery, variable half-lives, molecular degradation, or even sampling bias^19^. Additionally, certain factors specific to the current study design may also have contributed to this discordance in gene and protein expression. Firstly, unlike the transcriptomic analysis, proteomic analysis was not restricted to the cellular component of the TW sample, thus permitting the detection of secreted proteins. This approach was considered more likely to provide a more holistic assessment of the airway immune status as well as offering a greater potential to identify biomarkers with potential clinical and/or training applications. Secondly, gene expression data was obtained from each individual animal; whereas, protein analysis was conducted on pooled samples at each time point. This approach was justified on financial grounds due to the high cost associated with the application of these technologies. It should also be noted that, due to insufficient protein yield, samples from Horses 3, 7 and 12 were not included in the proteomic analysis for both time points. Thirdly, the lack of overlap in gene/protein expression in the current comparison can also be explained by the high ratio (approx. 27) between the expressed genes (21,357) and proteins (802) detected using the applied methodologies. Lastly, with such transcriptomic studies, the conclusions that can be derived from the data generated are highly dependent on the quality of the annotations of the genome used, which for the horse remains challenging^35^. Overall, consideration of these study-related factors and the recognised discordance between gene and protein expression does significantly question the validity of inferring changes in protein expression from transcriptomic data alone. Rather, this study highlights the value of applying a combined transcriptomic and proteomic approach when studying cellular mechanisms in both health and disease. Despite the lack of agreement at the individual gene and protein level, significant similarities were observed at a pathway and cascade level.

We demonstrated a clear modification of both mRNA and protein expression in TW derived samples during the training period. Based on the multiomics methodologies applied, we identified 2138 differentially expressed equine genes and 260 proteins during this period. Our findings are consistent with those derived from comparable studies in humans and horses^30,36,37^, and support a clear association between intense training and immune system deregulations, haemopoietic and metabolic abnormalities and cellular stress at the level of the airway. It is conceivable that such training-associated changes may play a role in increasing susceptibility to opportunistic infection and airway inflammation in racehorses.

### Local immune responses in tracheal airway immune cells during training

Analysis revealed the increased expression of several myeloid cell chemoattractants (e.g., *CCL2*, *CSF3R*, *S100A9* and other related S100 genes) at time point T1. Various relevant gene ontology terms were observed, such as neutrophil and monocyte chemotaxis, highlighting a level of immune cell activation in the airways. Additionally, acute phase response, cellular stress and oxidative phosphorylation also characterise the response to intense training.

Overall, both the transcriptomic and proteomic data were consistent with a level of inflammation in the airways during training, a phenomenon very well documented in racehorses. Based on the TW differential cytology results, 3 out of 16 animals developed airway neutrophilic inflammation (as defined by neutrophil ratio > 20) at time point T1, consistent with a low level of airway inflammation during training, despite the absence of clinical signs or evidence of poor performance. In light of the well-recognised association between intense exercise and an acute phase response in both humans and horses^38^, it is quite conceivable that repetitive periods of arduous exercise over a prolonged period of time may also result in low level airway inflammation. Indeed, recent equine studies have shown that an intense training programme will induce an “inflammation-like state” based on the measurement of acute phase protein SAA, which is greater than that induced by a lighter physical activity program^13^. Serum amyloid A was also significantly induced during the training period (T1) in our study. Despite the relatively low number of horses with a significant elevation in TW neutrophil ratio at T1, we did detect a training-associated upregulation of myeloperoxidase (MPO) protein expression, potentially reflective of a low level of airway neutrophil presence/activity below the threshold of detection by TW cytological examination. Indeed, MPO has previously been proposed as a more sensitive marker of airway neutrophil presence compared to cytology^39^. Despite its beneficial properties in host defence, overproduction of MPO may exert detrimental effects during inflammation^40^, including tissue damage and vascular dysfunction through the generation of potent ROS and activation of MMPs^40,41^. Consistent with these downstream effects, we also detected the upregulation of several MMPs (including *MMP9*) and ROS in association with training^42–44^.

In addition to inflammation, training was associated with evidence of tissue remodelling. C*hitinase 3 like 1, ARG2* and *MMP9*, all upregulated during training, have previously been linked to airway remodelling and/or declining lung function^45^. *MMP9* has been shown to contribute to airway remodelling in severe equine asthma^36^. Training was also associated with an upregulation of *ORMDL3* and *HLA*, both of which are highly associated with human and murine asthma^45^, with *ORMDL3* playing an important role in airway remodelling (increased airway smooth muscle, sub-epithelial fibrosis and mucus)^46^. *Interleukin 4 induced 1*, another gene which was highly expressed during training, has been shown to inhibit T cell proliferation, regulate the programming of macrophages towards a polarised M2 phenotype and downregulate LPS-induced *TNF* expression in murine macrophages^47^. Indeed, the data suggests that training may induce an overall immunomodulatory milieu at the level of the airway, characterised by increased expression of *IL4I1*^47^ and ROS^48^. This may explain the significant downregulation of *TNF* during the training period and the identification of gene ontology terms related with apoptosis and cell death, as ROS have been shown to regulate both necrotic and apoptotic T activated cell death via caspase activation^48^.

#### Training-associated IFN response on equine airways

A large number of IFN regulated and stimulated genes were differentially expressed during the training period. The IFN response is considered a key driver of inflammation in the lung by stimulating the recruitment and activation of immune cells, often in response to viral infection. Despite these beneficial effects, the negative regulation of the IFN response is necessary to avoid excessive and continuous activation of IFN-induced pathways with associated collateral tissue damage^23^. Moreover, there is recently increasing evidence that type I IFNs are not only induced by viral infections, but also by bacterial and fungal infections. Indeed, in the case of bacterial lung infections, the type I IFN mediated signalling may be detrimental^49^.

Training resulted in a greater differential expression of genes related to the type 1 IFN response, compared to type II and III IFN responses. Many of the recently described and highly conserved mammalian type I IFN ISGs were differentially expressed during training; these were related with antiviral activity, antigen presentation, PAMP detection and apoptosis. Interestingly, we identified several of the ISGs upregulated during training to be associated with ubiquitination; these included the ring finger proteins *RNF19A*, *RNF25*, *RNF181*, *RNF183* and highlighted protein reformation as part of the IFN response. This was also reflected in the pathway analysis, as protein synthesis was one of the top Biofunctions detected in our datasets. Recent findings derived from different mammalian species (including the horse), showed the type I IFN response to positively bias the sensitivity of RNA virus surveillance initiatives, recognising the problems associated with differentiating between “self” and “exogenous” cytoplasmic RNA^23^. Indeed, it is feasible that the type 1 IFN signature identified during training may be attributable to sub-clinical viral infection in this cohort of horses. Further work on a more geographically diverse population would be required to address this possibility. Despite the transcriptomic and proteomic evidence of altered immunity during the training period, the causal factors which underpin these changes remain speculative. Such causal factors may act in isolation or in concert and may include the following: repetitive periods of intense exercise, changes in airborne environment and subclinical infection. Indeed, both infectious and environmental causes have been proposed for the increased risk of airway inflammation within the early training period, potentially reflecting the co-mingling of horses from diverse locations and increased housing associated with the transition into this period^6,50–52^.

Previous studies have identified an association between the bacterial load within the trachea and both cytological and endoscopic evidence of airway inflammation^6,50,52^. Furthermore, in addition to the early training period, the detection of bacteria (streptococcal species) within the trachea was associated with evidence of airway inflammation; however, this latter association was not confirmed as causal, potentially reflecting a more indirect association (e.g. increased colonisation of mucus following compromised clearance). In our study, the timing of the second sample collection was such that this “early training” association should have subsided, which, together with the limited cytological or endoscopic evidence of significant airway inflammation offers more assurance that the transcriptomic and proteomic data was not solely reflective of the early transition to the training environment. Viral infections should also be considered as a potential explanation for the association between training and airway inflammation, particularly in light of the specific IFN pathways altered by training. Indeed, previous studies have shown that viral infections are common among young horses after entry into training^53,54^. However, attempts to identify viral RNA based on proteomic analysis, failed to identify any appropriate candidates. If a viral cause was indeed present, it was not associated with overt clinical signs, nor did it appear to impact athletic performance.

Previous studies have demonstrated a clear association between housing and airway inflammation, reflective of the increased exposure to organic dust^55,56^. As horses were housed during the training period and largely at pasture during the T0 sampling, it is quite feasible that some of the immunological changes observed, at both the individual gene and protein level and the pathway level, were consistent with an increased exposure to organic dust; however, this was not universally or consistently reflected in the TW differential cytology data. The ventilation within the stable was considered to be good and all horses were bedded on low dust shavings and fed haylage from the ground. Additional data relating to dust exposures would have assisted the interpretation of the data in light of the change in housing; however, this was not possible within the constraints of the study design. Lastly, in line with previous studies^10^, the immunological alterations associated with training may have been attributable to the repeatable episodes of high intensity exercise. Frellstedt et al. previously demonstrated training-associated changes in immunity at the level of the lower airways in treadmill exercised horses^10^.

### Training induced cellular stress

As well as the potentially direct effect of training on airway immunity, various transcription modulation processes also seem to play an important role. Cappelli et al. recently demonstrated the dominant role of transcription modulation in orchestrating the genomic response of equine PBMCs to exercise induced stress^57^. Muscle ATP demand increases with increasing exercise intensity with the rate of production dependent on the availability of oxygen, carbon substrates, Ca^2+.^and other molecules including, nitric oxide and reactive oxygen and nitrogen species (RONS)^58^. During intense exercise, increased RONS production may cause oxidative stress and damage to cellular structures and reduce mitochondrial efficiency, resulting in inflammation and transient immune dysfunction^59^.

In the current study, pathway analysis detected several differentially expressed genes related to oxidative stress, oxidative phosphorylation, mitochondrial dysfunction and protein synthesis (Supplementary Datas 4, 5). Reactive oxygen species modulator 1 gene, responsible for increasing the level of reactive oxygen species (ROS) in cells, was significantly upregulated and superoxide dismutases (SOD), the first line of defence against superoxide radicals^59^, were downregulated during the training period. At the protein level, both SOD1, with the capacity to limit the detrimental effects of ROS and apoptotic signalling, and SOD2 were significantly downregulated during the training period. Proteomic analysis also revealed downregulation of other antioxidants; these included thioredoxin (TXN) and various heat shock proteins (HSP90B1, HSPA5, ST13, HSPD1, HSPE1, HSPB1)^60^. *TXN* has the capacity to directly interact with several transcription factors including NFKB^60^, also regulated by ROS and which itself regulates the transcription of acute phase, cytokine and cell surface receptor genes; in some systems, antioxidants have been shown to reduce or block NFKB activation^60^. Our data showed the training-associated differential expression of several genes (*NFKBIZ*, *CARD19*, *NKAP*) involved in the NFKB pathway. ROS can also activate MAP kinases via a Ras-dependent mechanism^60^ and several components of the MAPK/ERK1/2 signalling pathway were either upregulated (*MCRIP2, LAMTOR1, LAMTOR2, LAMTOR4, MAPK13, MAPKAPK3*) or down regulated (*MAP3K7, GIMAP7, MAP3K8*) during the training period. The unchecked production of ROS and nitrogen reactive species will ultimately jeopardise cell survival^58^, a consequence consistent with the evidence of stress induced cell death detected in our dataset; namely, the training associated upregulation of members of the BCL2 family (*BAD*, *BAX*, *FADD*), *CARD19*, *AIFM2* and the pro-apoptotic molecules (*BAX*, *TMBIM1*). Excessive ROS production will also result in impaired mitochondrial efficiency, as detected during fatiguing and intense prolonged exercise in humans^58^. Canonical pathway analysis of both our transcriptomic and proteomic datasets revealed mitochondrial dysfunction in relation to training. The upregulated *COX14* gene encodes for a core protein component of the mitochondrial translation regulation assembly intermediate of cytochrome c oxidase complex (MITRAC) which is essential for the regulation of complex IV assembly of the mitochondrial respiratory chain. Upregulation of genes encoding several components of the complex IV (*COX5B*, *COX6A1*, *COX6B1*, *COX7A1*, *COX7A2*, *COX8A*) were also detected during the training period.

### Haemopoietic abnormalities during training

In light of the very high prevalence of EIPH in racing Thoroughbreds (approaching 100%)^18^, it is unsurprisingly that most of the horses (~ 69%, 11 out of 16) developed cytological evidence of EIPH during the training period. The pulmonary pathology associated with EIPH reflects changes in the pulmonary interstitium and vasculature, resulting in interstitial oedema and fibrosis^61,62^; yet, to date, none of the EIPH studies have examined the transcriptomics of airway derived cells harvested from cases. Analysis of gene expression data derived from the current study revealed minimal differences between EIPH affected (n = 11) and unaffected (n = 5) horses during the training period (data not shown); however, this has reflected the small group sizes following subdivision of the T1 cohort; however, several genes related to haemopoietic abnormalities were detected in both sub-groups, suggesting that bleeding may have occurred even in those cases with no clear cytological evidence of such. Our data does however support the potential value in expanding the transcriptomic analyses to a larger sample population in order to more critically assess the gene expression profile of EIPH cases.

### Transcriptomic profile of racehorses with high neutrophil count on TW samples

Although only a small number of horses developed a significant airway neutrophilia during the training period, supplementary comparative analysis was applied to the data derived from the three horses with the greatest magnitude of TW neutrophilia, compared with the other horses at time point T1. This revealed the differential expression of 57 genes, a number similar to that reported in circulating leukocytes derived from both Standardbred and endurance horses and BALF samples from horses with airway inflammation^63,64^. Also consistent with previous reports, almost all differentially expressed genes were upregulated, with only three being downregulated. The differential expression of *IL1RN* in the group of horses with airway inflammation (Supplementary Data 8), was consistent with the findings of Hansen et al. who reported an association between *IL1RN* expression in BALF derived cells and the neutrophilic form of MMEA^65^. Similarly, genes cysteine rich secretory protein LCCL domain containing 2 (*CRISPLD2)* and transglutaminase 3 (*TGM3)* were also present in BALF cells derived from horses with neutrophilic form of MMEA^64^. Interestingly, the neutrophilic group also showed increased expression of the mucin gene *MUC5AC*, shown previously to contribute to mucus hypersecretion in severe equine asthma^66^ and also highly expressed in bronchial biopsies from asthmatic humans^67^. Overall, the transcriptomic changes in this small group of horses were consistent with the airway cytological profiles observed, reflecting pathways associated with neutrophil chemotaxis, response to stress and immune defence^36^. Interestingly, prior comparative transcriptomic analyses of endotracheal biopsies derived from asthmatic horses also revealed the differential expression (compared with non-asthmatic horses) of a similar gene list to that identified in the current study. This gene set included *CSF3R*, *CXCR2*, *PLEK*, *IL1RN*, *RETN*, *VSTM1*, *TREM1* and *PLAUR*, and is related to neutrophil chemotaxis and immune responses. Colony stimulating factor 3 is responsible for neutrophil production and proliferation and promotes neutrophil trafficking by modulating chemokine and adhesion receptors, such as CXCR2 that mediates neutrophil migration to sites of inflammation. Although difficult to detect in blood, CSF3 is secreted to a greater extent during infectious or inflammatory conditions^68–70^ including human asthma where it was positively associated with BALF neutrophil numbers and sputum from asthmatic patients^67^. Moreover, CSF3 is considered as a potential target against pathological inflammation and tissue remodelling occurring in human asthma^67^. Other genes detected in this group, such as *TREM1* and *PLAUR* have also been reported in human asthma^45,71^.

A number of Th1 immune response agents were also enriched in the neutrophilic group of horses (*CXCR2*, *IDO1*, *CSF3R*, *ISG20*, *IL18RAP* and *TREM1*). Thus, it appears that the equine airway neutrophilia detected in these cases was associated with activation of the innate immune system, with a likely Th1 polarised response. Similar observations have been previously reported, although, within the context of MMEA, suggesting that immunological pathways vary according to the cytological profile of the airway inflammatory response and potentially the phase of the disease^72^.

Despite limitations of the current study, this is the first whole genome sequencing study performed on equine TW derived cells. Results shared important similarities with previous equine and human studies and although the aim of the study was focussed on the impact of training, transcriptomic analysis detected a number of novel genes potentially related to disease pathogenesis which may provide new insights for future studies. It remains feasible that the assessment of gene expression in TW-derived cells may represent a relatively non-invasive method to identify molecular endotypes of equine asthma in larger studies. However, detailed phenotypic characterisation of the horses included in these studies would be crucial.

## Conclusions and future perspectives

This study has shown for the first time the feasibility of performing transcriptomic and proteomic analyses on TW samples collected from horses and, despite certain inherent study limitations, demonstrated significant training-associated alterations in both gene and protein expression within these samples. Furthermore, the current study improved our understanding on the respiratory immune mechanisms associated with airway inflammation, which has recently been recommended as an important research priority^4,7,73^. Alterations in gene and protein expression are particularly related to airway immunity, haemopoietic abnormalities, oxidative and cellular stress and the results indicate the inflammatory status of the airway in association with training, despite not being cytologically apparent. Overall, the datasets described here represent the most comprehensive results related to the response of airway immunity of the horse to intense training and could open doors for future research on equine exercise immunology based on a wider, better defined and preferably naïve population entering training for the first time. Developing such novel information could have a significant positive impact on Thoroughbred welfare and the racing industry.

Although this study focussed on the temporal changes associated with entry into a training program, the combined –omics approach could also provide a more definitive and comprehensive characterization of lower airway diseases. Although, in this respect, further work comparing TW and BALF samples, similar to that already reported in relation to cytological correlations, is warranted. Finally, as previously suggested, many similarities were observed with human-derived data in relation to exercise immunology, further supporting the use of the horse as an attractive animal model from which translational application of findings to humans may be justified.

## Methods

### Animals used in the study

Tracheal secretions were collected from 16 male Thoroughbred horses [6.9 ± 0.5 years (mean ± SEM)]. All horses were housed at the same racing yard and were trained by the same trainer. Samples were obtained as part of a routine assessment of respiratory health (differential cytology) and residual sample was retained for the ongoing proteomic and transcriptomic study. The use of residual sample material was approved by the Veterinary Ethical Review Committee of the School of Veterinary Medicine, University of Edinburgh and informed consent was obtained from the trainer, under who’s care the horses in the study were maintained. Enrolled horses underwent physical examination, endoscopy of the respiratory tract, and TW. All horses were clinically healthy and treated according to standard welfare procedures. All methods reported are in accordance with ARRIVE guidelines (https://arriveguidelines.org)^74^. All experimental protocols were approved by Ethical Review Committee of the School of Veterinary Medicine, University of Edinburgh, following the relevant guidelines and regulations relating to the provisions of the Animals (Scientifc Procedures) Act 1986. All methods were carried out in accordance with relevant guidelines and regulations.

### Sample collection and cell isolation

#### Field work—tracheal wash sample collection

Samples were collected at two different time points: T0, when the horses were at rest between racing seasons and T1, when the horses were in active race training. T0 sampled were collected following 59 ± 5.7 days at rest and T1 samples were collected when the horses were considered “racing fit”, having entered into the training program for approximately 2.5 months (76 ± 11.4 days) and having already ran in at least 2 competitive races (average number of starts 3.9 ± 0.4) that season. All TW samples were collected using a trans-endoscopic technique, as previously described^27^. Horses were manually restrained and a nose twitch applied when necessary. A 1500 mm working length, 9.2 mm outer diameter video endoscope (2.8 mm biopsy channel) (Aohua, China), was passed via the ventral nasal meatus, via the pharynx and into the tracheal lumen via the *rima glottidis*. Following assessment of the amount and nature of mucus deposits within the tracheal lumen, approximately 20 mL of sterile 0.9% saline at room temperature was instilled via a catheter passed via the biopsy channel of the endoscope at the proximal aspect of the trachea. The endoscope was then further advanced to the level of the thoracic inlet where the pool of instilled fluid had gravitated. As much fluid as possible was subsequently aspirated via the transendoscopic catheter. Samples were stored on ice and submitted for laboratory analysis at the Roslin Institute and the Royal (Dick) School of Veterinary Studies and processed within 4 h of collection.

#### Differential cell count of tracheal wash

An aliquot of 0.5 mL was submitted to the pathology lab at the Royal (Dick) School of Veterinary Studies for differential cell count analysis (DCC). Differential leucocyte count (minimum of 200 cells) was performed on dithiothreitol (DTT) treated samples and expressed as a percentage of total non-squamous and non-epithelial nucleated cells, as previously described^17^. Horses were considered free of inflammatory airway disorders based on the following DCC cut off values: neutrophils < 20%, eosinophils (< 1%)^26,27^. Samples containing haemosiderophages were considered consistent with a diagnosis of EIPH^18^.

#### Total cell isolation from tracheal wash

A 1 mL aliquot of sample was stored at − 80 °C for future proteomic analyses and the remaining sample incubated for 15 min at room temperature in 0.1% DTT to depolymerize secreted mucin, as previously described^17^. Dithiothreitol has been demonstrated to cause no deleterious effects on human sputum derived cells or interfere with surface marker measurements using flow cytometry^75,76^. Following gravity filtration through a 100 µm pore mesh filter, the sample was centrifuged at 400×*g* for 10 min at 4 °C. Supernatant was carefully removed and the cell pellet re-suspended in Dubelcco’s PBS, from which a total cell count (excluding squamous epithelial cells) and cell viability (Trypan Blue exclusion staining) was performed using a haemocytometer. Afterwards, the cell pellet was resuspended in 1 mL of Trizol and stored at − 80 °C for future RNA analysis.

### RNA analysis

#### Total RNA extraction

Total RNA was extracted using a combination of Trizol reagent (Thermo Scientific™, 15596026) and an RNAeasy plus micro kit (Qiagen, cat no 74034), according to manufacturer’s instructions. Following removal of the supernatant, the remaining cell pellet was lysed by the addition of 1 mL Trizol Reagent. Subsequently, 0.2 mL 1-Bromo-3-chloropropane (BCP) (Sigma Aldrich, cat no B9673-200ML) was added per 1 mL of Trizol. The sample was shaken vigorously for approximately 30 s and left at RT for 5 min to allow complete dissociation of nuclear-protein complexes. The homogenate was then centrifuged at 18,000×*g* for 15 min at 4 °C resulting in the formation of a lower red phenol–chloroform phase, an interphase, and an upper colourless aqueous phase. The aqueous phase contained the RNA and had almost 50% of the volume of the Trizol used, plus the volume of the sample. Following transfer to a clean tube for the precipitation step, 0.5 mL of 70% ethanol was added, the sample stored for 2 h at − 20 °C and then transferred to an RNeasy spin column and centrifuged at 18,000×*g* for 5 min at 4 °C. Following centrifugation, the flow through was removed, the RNA washed once with RW1 buffer and DNA treatment was performed using the RNase-Free DNase Set (Qiagen, cat no 79254) according to the manufacturer’s instruction. Due to DNA contamination, this step was performed twice or samples were run through gDNA Eliminator Spin Columns twice after the elution step. Afterwards, the RNA membrane was washed with RW1, RPE and 80% ethanol. Finally, RNA was eluted in 15 µL RNase-free water and RNA samples were stored at − 80 °C until further use.

#### RNA quality assessment

RNA concentration and purity was measured using ND-1000 Nanodrop spectrophotometer (Thermo Scientific, Wilmington, USA) by measuring absorbance at 260 and 280 nm (A260, and A280 respectively). Purity of RNA was determined using the A260/A280 ratio; a ratio approximating 2 was considered to be indicative of pure RNA. RNA integrity was confirmed with the High Sensitivity RNA ScreenTape system (Agilent Technologies). A RNA integrity number (RIN) greater than 7 was considered appropriate for qPCR and RNAseq analysis.

#### RNA sequencing analysis

Total RNA was processed using NEBNext Ultra Directional RNA Library Prep Kit by Novogene (Novogene Europe) to generate cDNA libraries according to the manufacturer’s instructions and subsequently sequenced with an Illumina NovaSeq 6000 platform (Illumina, San Diego, USA) at a depth of 35 M reads strand-specific 150-bp paired-end per sample. Ribosomal RNA (rRNA) was depleted from samples for total RNAseq, using biotinylated, target-specific oligonucleotides with Ribo-Zero rRNA removal beads.

#### Processing of next-generation sequencing data and differential expression analysis

Raw data is deposited in the Gene Expression Omnibus under the study accession number GSE181221 (https://www.ncbi.nlm.nih.gov/geo/). For each sample, a set of expression estimates, as transcripts per million (TPM), were obtained using the high speed transcript quantification tool Kallisto^77^ as previously described^78,79^. Raw sequencing reads were processed to remove adaptors and poor-quality sequences (Q25 and below) using the Cutadapt program^80^. Quality control of trimmed data was assessed using FASTQC (https://www.bioinformatics.babraham.ac.uk/projects/fastqc/) and Multiqc^81^. Kallisto (0.46.0.1) was ran on all samples, using as its index the complete set of 59,087 predicted transcripts for the EquCab3.0 genome (ftp://ftp.ensembl.org/pub/release-99/fasta/equus_caballus/cdna/Equus_caballus.EquCab3.0.cdna.all.fa.gz) and the quantification of gene expression was calculated. The horse annotation (GTF) was obtained from Ensembl (EquCab3.0) software. For the differential analysis of count data, we used the DESeq2 method which integrates methodological advances with novel features to enable a more quantitative analysis of comparative RNAseq data using shrinkage estimators for dispersion and fold change^31^. Differentially expressed genes were then identified by applying an FDR cut off of 0.05. Analysis was performed in R *v 3.5.0* (https://www.r-project.org/).

#### Quantitative PCR (qPCR)

One microgram total RNA was converted to complementary DNA (cDNA) using the prescription NanoScript reverse transcription kit (SuperScript III First-Strand Synthesis System, Invitrogen, Cat No 18080051), according to the manufacturer's instruction. cDNA was stored at − 20 °C until further use. Transcript levels were quantified in triplicate using an MX3005P quantitative polymerase chain reaction (qPCR) system (Stratagene) with primers listed in Supplementary File 2 and qPCRBIO SyGreen Mix Lo-ROX kit (PCRBIO). Primer efficiency was validated with a standard curve of five serial dilution points and *SDHA* was used as a reference gene. SDHA was used as a reference gene because it remained stable in the RNAseq data and it has previously been determined to be the most stable housekeeping gene to study equine exercise-induced stress^64,82^. Reverse transcriptase negative and “no template” control samples were included in each run as negative controls. Data were analysed using Stratagene MxPro software and relative gene expression was calculated using the 2^−ΔΔCT^ method^83^.

### Proteomic analysis

#### Protein extraction

Briefly, following sonication, an aliquot of 500 µL of untreated TW sample was concentrated to 300 µL using a SpeedVac Vacuum Concentrator, in order to increase protein yield. Samples were homogenized in label-free buffer (100 mM Tris, ph 7.6 and 4%w/v SDS) + 1% Halt Protease Inhibitor Cocktail, EDTA-Free (Thermo Scientific, cat no 87785). Following homogenization, samples were centrifuged at 20,000×*g* for 20 min at 4 °C. The supernatant containing the solubilized protein was removed and stored at − 80 °C. Protein concentration of samples was determined using a Micro BCA Protein Assay Kit (Thermo Scientific™, cat no 23235) according to the manufacturer’s instructions. Finally, total protein analysis was carried out for quality control purposes and to determine equivocal protein load between samples. Samples were separated by electrophoresis on gradient gels (NuPAGE 4–12% Bis–Tris Protein Gels, 1.0 mm, 12-well, Fisher Scientific, cat no NP0322BOX) and stained with InstantBlue™ Protein Stain (Expedeon Ltd, cat no ISB1L) as previously described^84^. The stained gel was then imaged using the LICOR Odyssey imager to visualise and quantify the total protein load within each lane of the gel using associated Image Studio Software.

#### Sample preparation for LC–MS

Samples of animals collected at T1 and T0 were pooled and analysed in the Mass Spectrometry Facility at the Roslin Institute (https://www.ed.ac.uk/roslin/facilities-resources/proteomics-and-metabolomics-facility). Note samples from Horses 3, 7 and 12 were not included in the proteomic analysis for time point T0 and T1, because insufficient protein material was isolated. The protein samples (T0 and T1) were then reduced with dithiothreitol and alkylated with iodoacetamide prior to tryptic digestion on S-TRAP (Protifi, USA) cartridges following the manufacturer’s protocol. The resulting peptides were cleaned up and labelled by reductive di methylation on Solid Phase Extraction cartridges.

Stable isotopic reductive dimethylation of the resulting peptides was performed on OASIS HLB columns (Waters) following standard methods^85^. The peptides were acidified by addition of trifluoroacetic acid to 0.1% (v/v) before loading onto conditioned OASIS HLB columns. Peptides from T0 and T1 samples were labelled with ‘light’ and ‘heavy’ labelling reagents, respectively. The ‘light’ buffer consisted of 0.8% (w/v) formaldehyde and 0.12 M sodium cyanoborohydride carrying hydrogens in their natural isotopic distributions in triethyl ammonium bicarbonate (TEAB) buffer. ‘Heavy’ buffer consists of 0.8% (w/v) deuterated formaldehyde and 0.12 M sodium cyanoborohydride in TEAB buffer. The bound peptides were washed and eluted with 0.1% (v/v) trifluoroacetic acid in 70% (v/v) acetonitrile. The eluted peptides were concentrated using a speed vacuum concentrator. The labelled ‘light’ and ‘heavy’ peptides were mixed together before fractionation by strong cation exchange chromatography on a Polysulphoethyl A column (PolyLC Inc USA). Fractions were desalted on Stage-Tip columns^86^. Each fraction of peptides was loaded onto an Acclaim PepMap100, C_18_, 3 μm, 100 Å, 75 μm (internal diameter) × 50 cm column using an UltiMate RSLCnano LC System (Dionex).

#### LC–MS analysis

The peptides eluted by reversed-phase chromatography were analysed by a micrOTOF-Q II mass spectrometer (Bruker) following earlier method with minor modifications^87^. Spectral data were processed by PoteinScape server 3.1 (Bruker) software and the resulting peak lists were searched using Mascot 2.4 server (Matrix Science) against Uniprot Horse (Equus Caballus) sequence database containing 44,472 entries (https://www.uniprot.org/proteomes/UP000002281). Carbamidomethylation of cysteine was used as a fixed modification and oxidation of methionine and light and heavy dimethylation of N-terminus and lysine were chosen as variable modifications. False discovery rate was limited to < 1% for peptide IDs after searching decoy databases. Dimethyl quantification was performed by using WARPLC plugin (Bruker) on Proteinscape 3.1 server to integrate extracted ion chromatogram of every precursor. Peptide ratios were normalized based on setting overall peptide median ratio at 1, which corrects for unequal protein sampling and a coefficient of variability of peptide ratios were also determined for each quantified proteins.

### Gene ontology and pathway analysis

Identification of enriched KEGG pathways in the upregulated and downregulated gene lists was performed with DAVID (v6.8)^20^. Pathway analysis was performed using Ingenuity Pathway Analysis^21^ to infer the functional roles and relationships of the differentially expressed genes based on the log2 fold change value of each gene.

### Statistical analysis

Descriptive statistics were performed using R *v. 3.5.0* (https://www.r-project.org/). After testing for normality, a one-sample t-test was employed to identify differences in cells between time point T0 (rest) and time point T1 (training). Statistical significance was assumed at p < 0.05. Numeric results are presented as mean ± SEM.

## Supplementary Information

Supplementary Information 1.

Supplementary Information 2.

Supplementary Information 3.

Supplementary Information 4.

Supplementary Information 5.

Supplementary Information 6.

Supplementary Information 7.

Supplementary Information 8.
