# Supplementary material for: Training associated alterations in equine respiratory immunity using a multiomics comparative approach
Source: Sci Rep. 2022 Jan 10;12:427. doi: 10.1038/s41598-021-04137-3 (PMC8748960; doi:10.1038/s41598-021-04137-3)
Supplement: Supplementary file 2 — Supplementary Information 2. [file 41598_2021_4137_MOESM2_ESM.docx]

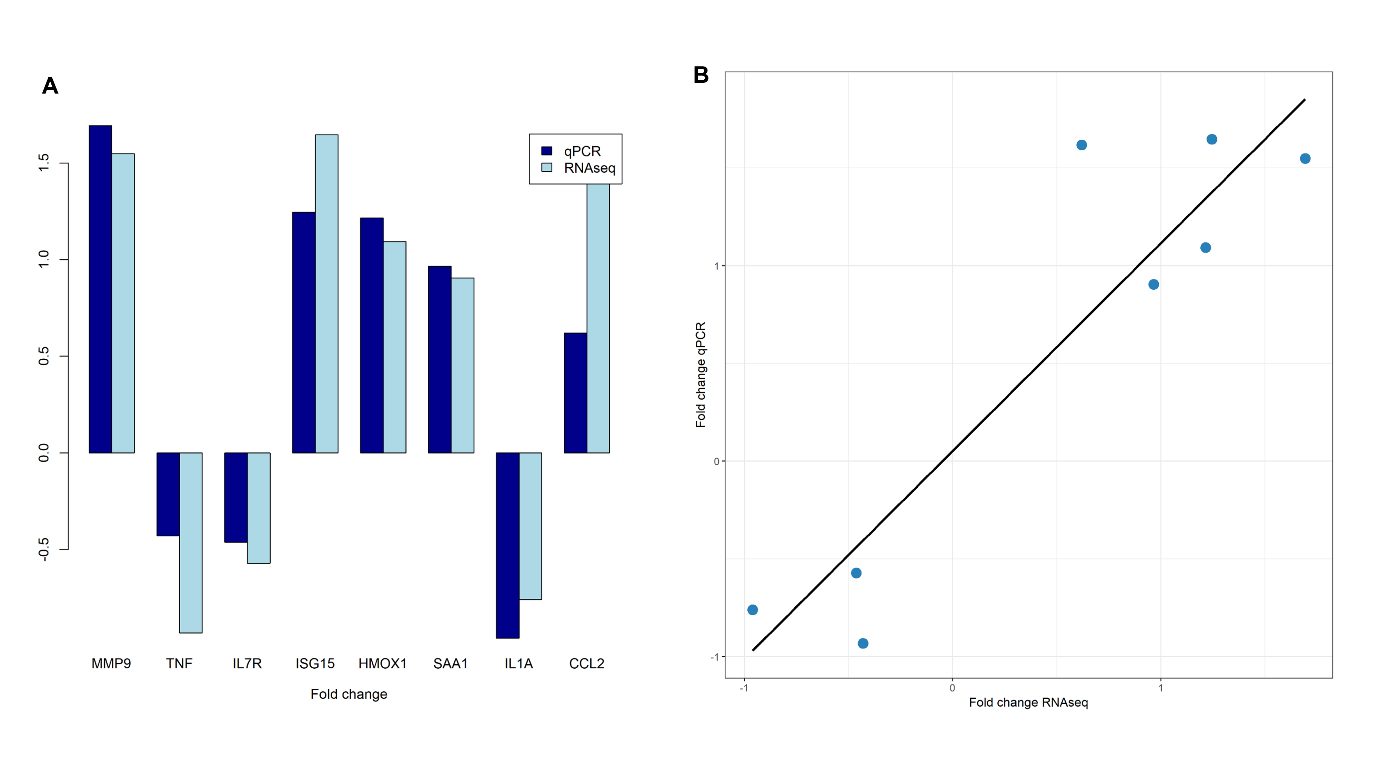


***Expression profile of RNAseq and qPCR data.*** *Expression profile of RNAseq and qPCR data for selected genes were compared using the same samples.* **A)** *The data are consistent with the results of RNA-seq analysis for these genes in the same samples, where MMP9, ISG15 and HMOX1 were upregulated and TNF and IL7R were downregulated. Expression was normalized using SDHA* *as a reference gene in qPCR. Results represent mean values and are expressed as Log2 values of the fold change* and are representative of five biological replicates. **B)** *RNA-seq and qPCR Log2 values of the fold change were significantly correlated with a correlation coefficient r = 0.92 (Pearson correlation test) and P value = 0.004).*

***Primers used in this study***

| **Target** | **Primer orientation** | **Primer Sequence (5’-3’)** | **Size (bp)** |
| --- | --- | --- | --- |
| TNF | F | ATCTACTCCCAGGTCCTCTTC | 129 |
|  | R | CGTGTTGGCAAGGCTCTT |  |
| MMP9 | F | TTGACTCCACAGTGAACGGG | 120 |
|  | R | CAGGCTGTATCCTTGGTCGG |  |
| IL7R | F | TCCTCCGCAACTAGATGATTC | 109 |
|  | R | CTCTCCTCCGAAAGTTTTTGG |  |
| ISG15 | F | CAGTTCTGGCTGACTTTCGA | 102 |
|  | R | CAGGCGCAAGTTCATGTACA |  |
| HMOX1 | F | TGGCTTCTTCCTTTGGGCAT | 108 |
|  | R | TGCTTGTTGGTTGGGGAAGA |  |
| SAA1 | F | CCTGGGCTGCTAAAGTCATC | 127 |
|  | R | AGGCCATGAGGTCTGAAGTG |  |
| IL1A | F | ATGAGGATCGTCAACCACCA | 119 |
|  | R | TTCACTGCGTCGTCCAGATT |  |
| CCL2 | F | CAACAACTCTCAGGCCGAA | 262 |
|  | R | ATCTCCTTGGCCAATATGGTCT |  |
| SDHA | F | GGACAGAGCCTCAAGTTTGG | 116 |
|  | R | GTATCATATCGTAGAGACCTTCCATA |  |
